# Progressive Failure Characteristics of Brittle Rock under High-Strain-Rate Compression Using the Bonded Particle Model

**DOI:** 10.3390/ma13183943

**Published:** 2020-09-06

**Authors:** Xiaolin Huang, Shengwen Qi, Bowen Zheng, Songfeng Guo, Ning Liang, Zhifa Zhan

**Affiliations:** 1 Key Laboratory of Shale Gas and Geoengineering, Institute of Geology and Geophysics, Chinese Academy of Sciences, Beijing 100029, China; huangxiaolin@mail.iggcas.ac.cn (X.H.); zhengbowen@mail.iggcas.ac.cn (B.Z.); guosongfeng@mail.iggcas.ac.cn (S.G.); liangning@mail.iggcas.ac.cn (N.L.); 2 Innovation Academy for Earth Science, Chinese Academy of Sciences, Beijing 100029, China; 3 College of Earth and Planetary Science, University of Chinese Academy of Sciences, Beijing 100049, China; 4 China Highway Engineering Consultanting Corporation, Beijing 100089, China; zhanzhifa100@126.com

**Keywords:** brittle rock, high strain rate, progressive failure characteristics, microcracking, brittleness-to-ductility transition, ‘X’-shape failure, bonded particle model

## Abstract

This paper microscopically investigated progressive failure characteristics of brittle rock under high-strain-rate compression using the bonded particle model (BPM). We considered the intact sample and the flawed sample loaded by split Hopkinson pressure bar respectively. Results showed that the progressive failure characteristics of the brittle rock highly depended on the strain rate. The intact sample first experienced in microcracking, then crack coalescing, and finally splitting into fragments. The total number of the micro cracks, the proportion of the shear cracks, the number of fragments and the strain at the peak stress all increased with the increasing strain rate. Also, a transition existed for the failure of the brittle rock from brittleness to ductility as the strain rate increased. For the flawed sample, the microcracking initiation position and the types of the formed macro cracks were influenced by the flaw angle in the initial stage. However, propagation of these early-formed macro cracks were prohibited in the later stages. New micro cracks were produced and then coalesced into diagonal macro cracks which could all form ‘X’-shape failure configuration regardless of the incline angle of the flaw. We explored micromechanics on progressive failure characteristics of the brittle rock under dynamic loads.

## 1. Introduction

Some natural and man-made events often involve high-strain-rate loads such as strong earthquakes and engineering blasting. Understanding failure characteristics of the brittle rock under dynamic loads is of great importance for the rock engineering. A rock mass often bears a strain rate ranging from ~10 s^−1^ to ~10^2^ s^−1^ under seismic or blasting stress waves [1,2]. Numerous tests showed that the brittle rock was a highly rate-dependent material, of which the strength and fragment degree significantly increased with the strain rate increasing [3,4,5,6,7,8]. Meanwhile, the brittle rock is a discontinuous material, which often contains a great number of multi-scale flaws after complex diagenesis and tectonism. Due to the stress concentration effect, these flaws significantly influenced failure characteristics of the brittle rock [9,10,11,12]. A series of experimental studies were conducted to investigate the cracking behavior of the prism pre-cracked specimens of brittle rocks and rock-like materials under quasi-static uniaxial compression in previous studies. It was observed that wing cracks first appeared in the vicinity of flaw tips and then two types of secondary cracks occurred successively, and the trace of the wing crack was eventually parallel with the direction of the maximum principle stress [13,14,15]. Nevertheless, the cracking behavior of the brittle rock significantly changed under the high strain rate. By the split Hopkinson pressure bar (SHPB) system, many researchers explored dynamic fracturing characteristics of brittle rock using notched semi-circular bend specimens and found that both the fracture toughness and fracturing velocity increased with an increase in the loading rate [3,4,16,17,18]. Moreover, some scholars found that the cracking trajectory of the flawed rock sample mainly exhibited an ‘X’ shape, which was distinct from the quasi-static case [19,20].

Above studies mainly investigated failure characteristics of the brittle rock under dynamic loads from a macroscopic viewpoint. However, numerous studies showed that the failure of the brittle rock was a progressive process and experienced the evolution from micro cracks to macro cracks [21,22,23,24,25,26,27,28]. Reference [20] observed that micro cracks first appeared in the vicinity of flaw tips of the Carrara marble and gradually accumulated before the appearance of macro cracks under dynamic compression. Due to the difficulty in tracing the evolution of micro cracks inside rock, the experimental method is usually limited to investigate the progressive failure of the brittle rock. Thus, the numerical method is an alternative to feasibly solve this problem. In previous studies, the bonded particle model (BPM) based on the discrete element method (DEM) was widely used to reproduce the progressive failure behavior of the intact rock [29,30,31,32,33,34] and the rock containing flaws under quasi-static loads [35,36,37]. These numerical results were in line with experimental results, which indicated that the BPM was able to simulate the progressive failure of the brittle rock. Out of the quasi-static problem, the BPM was also applied to investigate dynamic-induced failure characteristics of the brittle rock such as the dynamic uniaxial compression strength [38,39,40], the Brazilian tensile strength [41], the dynamic mode-I fracture toughness [42,43], and the progressive fracturing of pre-cracked Brazilian disc specimens [44]. However, further studies are still required to clarify the progressive failure process of the brittle rock under dynamic loading, i.e., the effect of the strain rate on different types of micro cracks and their evolutions to macro cracks, and the ‘X’-shape failure configuration of the brittle rock containing an open flaw. The purpose of this study is to understand the progressive failure of the brittle rock under high-strain-rate compression using the BPM.

This paper was structured as follows. Section 1 reviewed previous studies on the progressive failure of the brittle rock and analyzed the limitations in these studies. Section 2 introduced the numerical models on the dynamic compression of the brittle rock. Section 3 showed the results in detail and these results were discussed in Section 4. Conclusions were given in Section 5.

## 2. Materials and Methods

**Figure 1:**
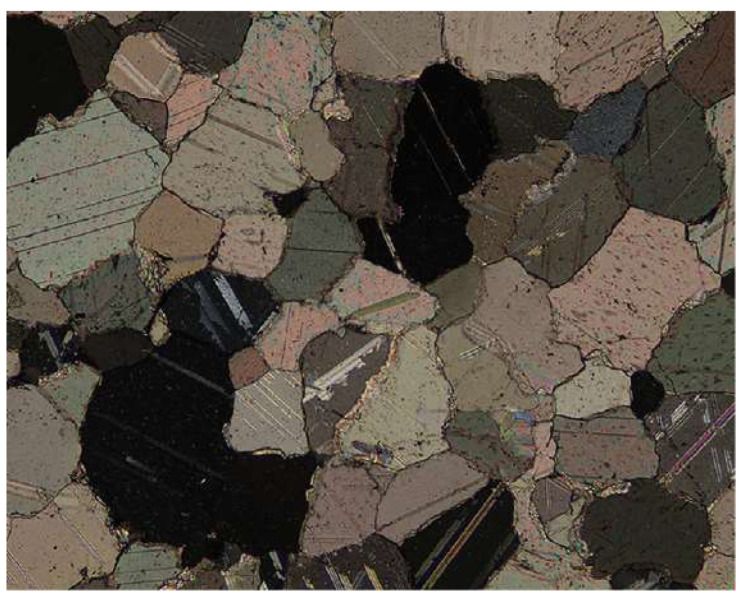
Polarized thin section of Carrara marble adapted from [47]. The width and height of the sections are 1.38 mm and 1.1 mm, respectively.

**Figure 2:**
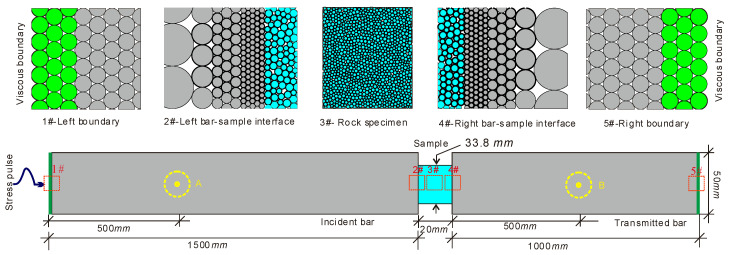
Numerical SHPB system based on the BPM. For interpretation of the references to color in this figure, the reader is referred to the electronic version of this paper.

In practice, the SHPB system has been widely adopted to test dynamic properties of geotechnical materials. Based on the BPM, many researchers simulated SHPB tests on filled joints and brittle rocks [39,40,42,44,45]. In the current study, the numerical SHPB based on BPM was also established to investigate the progressive failure characteristics of the brittle rock under the high-strain-rate compression. We adopted the experimental results of Carrara marbles by [20] for calibration and comparison. Carrara marble is a typical brittle and popular testing material. It originates from Italy, consisting of 99% calcite crystals (CaCO_3_) and 1% organic impurities. Due to low intrinsic crack density, fine crystal and low porosity, it has been widely used to conduct rock mechanics tests over the past decades [20]. Even though Carrara marble is one of the metamorphic rocks, its microscopic structure is granular as shown in Figure 1. Thus, Carrara marble is suitable for the simulation based on DEM. The numerical simulation was implemented by the software Particle Flow Code [46]. Three assemblies of bonded particles were used to simulate the incident bar, transmitted bar, and the rock sample as shown in Figure 2. There are two types of bonds in BPM, i.e., the contact bond and the parallel bond. For the contact bond, two adjacent contact particles can rotate relatively to each other which cannot bear the moment. For the parallel bond, two adjacent contact particles can suffer from the moment which cannot rotate relatively to each other. For the rock material, particles or grains are often interlocked. Thus, the parallel bond was applied in the current study.

We first considered the model of the intact rock as shown in Figure 2. The model consists of the incident bar, the rock sample, and the transmitted bar. Both the incident bar and the transmitted bar has a width of 50 mm but different length of 1500 mm for the incident bar and of 1000 mm for the transmitted bar. The intact rock sample has a length of 20 mm and height of 38.4 mm. Because the SHPB was made from steel, we adopted hexagonal close-packed particles with a fixed radius to build the incident bar and the transmitted bar. The contact among the sample and the bars were treated as interfaces without bonded strength and friction. Considering the accuracy and computation efficiency simultaneously, both the incident and transmitted bar was made up of particles with a fixed radius of 2.0 mm. Particles in the rock sample have random distributions on both the position and radius. The radius of the particle in the sample ranges from 0.08 to 0.128 mm. Note that there exist radius differences between the bar and the rock sample. To eliminate this defect, the particle radius in the incident bar gradually decreased near the sample and finally matched with the particle radius of the rock sample, which is around 0.12 mm (see 2# in Figure 2). Similar treatment was conducted near the right side of the rock sample (see 4# in Figure 2).

**Figure 3:**
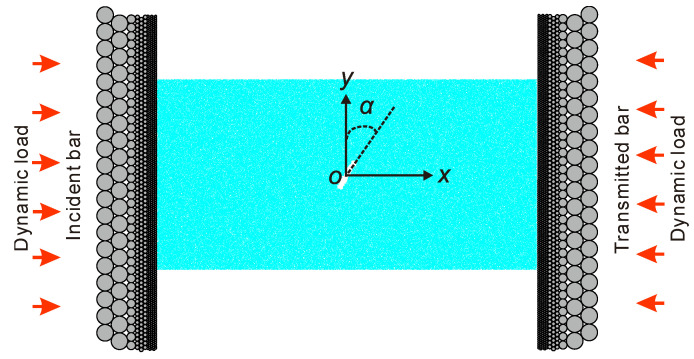
Sample containing an open flaw. For interpretation of the references to color in this figure, the reader is referred to the electronic version of this paper.

Besides the intact sample, we also considered the sample containing an open flaw as shown in Figure 3. The sample has a length of 60 mm and a height of 30 mm. The flaw has a length of around 5 mm and a width of around 1 mm, which was formed by deleting particles. The midpoint of the flaw overlaps with the geometry center of the sample. The long axis of the flaw has the angle of *α* with respect to the *y*-axis.

A practical SHPB system often induces approximately half-sinusoidal stress waves when a striker bar impacts pulse shaper mounted on the end surface of the incident bar. For simplicity, we directly input the stress wave at the left end of the incident bar in Figure 2 rather than used the strike bar during the simulation. Two strips of particles (three layers for each) with a green color were identified as the left and right boundaries of the model as shown in Figure 2. During simulation, these two boundaries were set as viscous boundaries to avoid the interference of reflected waves. The incident compressive stress wave was normally applied at the left boundary. The input waveform was a half-sinusoidal wave as

(1) σ(t)=Asin(2πft) 0≤t≤1/(2f),

where *A* denotes the amplitude of the stress wave; *f* is the frequency; *t* is the time. By adapting *A* or *f*, we can achieve different strain rates applied to the sample.

According to the BPM theory, a stress wave cannot be directly input to the boundary, which should be transformed into force and applied to each particle on the boundary. The method to apply the stress wave and to realize the viscous boundary can be seen in [45,48]. Upper and lower boundaries of the incident and transmitted bars were set as free boundaries. Two measure circles A and B with a same radius of 25 mm were set to monitor reflected and transmitted wave signals respectively. Centers of circles A and B were both located in the horizontal symmetry axis. The distance from the center of the circle A to the left end of the incident bar is 500 mm. The center of the circle B was located in the middle of the transmitted bar.

Based on the SHPB theory [49], the dynamic stress–strain curve of the sample can be calculated according to the recorded incident, reflected, and transmitted waveforms if stresses at the front and rear interfaces keep balance. According to the one-dimensional stress wave theory, we can determine the histories of the compressive strain *ε(t)*, the strain rate $\dot{\varepsilon }(t)$ and the compressive stress *σ(t)* within the sample by following equations.

(2) $$\varepsilon (t)=\frac{C}{L}\int_{0}^{t}\left(\varepsilon_{inc}(t)-\varepsilon_{ref}(t)-\varepsilon_{tra}(t)\right)dt,$$

(3) $$\dot{\varepsilon }(t)=\frac{C}{L}(\varepsilon_{inc}(t)-\varepsilon_{ref}(t)-\varepsilon_{tra}(t)),$$

(4) $$\sigma (t)=\frac{S_{b}}{2S}E(\varepsilon_{inc}(t)+\varepsilon_{ref}(t)+\varepsilon_{tra}(t)),$$

where, *S_b_* and *S* are cross-sectional areas of the bar and the sample respectively; *C* is the longitude wave velocity of the bar; *L* is the initial thickness of the sample; *E* is the Young’s modulus of the bar; subscripts “*inc*”, “*ref*”, and “*tra*” denote the incident, reflected and transmitted waves, respectively.

In the BPM, the synthesized macro-scale material behavior is related to micro-scale components. However, it is often difficult to determine micro properties such as the contact stiffness, the bonded strength and the friction so that the behavior of the BPM resembles that of the real material, and input properties of microscopic constituents are usually unknown. A feasible method is to forward fit the micro parameters by trial and error until the numerical simulation result matches well with the experimental data of the Carrara marble.

## 3. Results

### 3.1. Case of the Intact Sample

**Figure 4:**
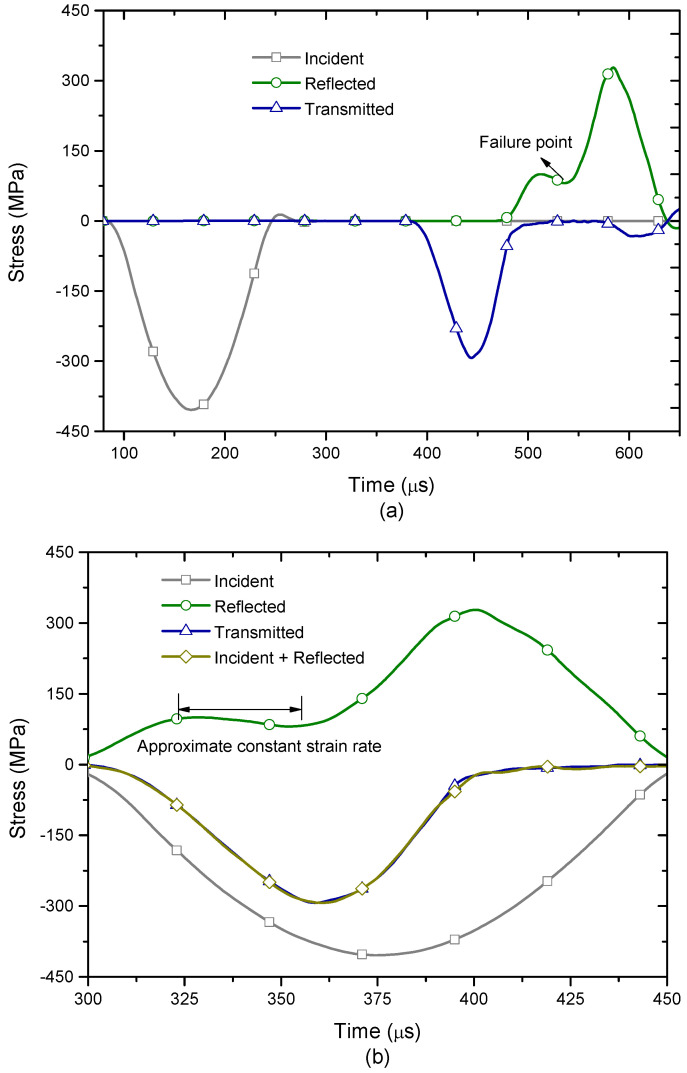
Stress waveforms recorded in the model. (**a**) The incident and reflected stress waveforms as well as transmitted waveforms through the sample; (**b**) stress balance check at the front and rear interfaces of the sample. For interpretation of the references to color in this figure, the reader is referred to the electronic version of this paper.

This section mainly focuses on the progressive failure process of the intact rock under high-strain-rate compression. The input stress wave has a fixed frequency of 3.333 kHz and varied amplitudes respectively. Table 1 lists the calibrated micro parameters for the steel incident and transmitted bars. Because bars cannot fail, the bonded strength was set as very large values. Table 2 lists the comparison of macro parameters between the BPM and the steel bar. Table 3 lists the fitted micro parameters for the Carrara marble. Figure 4 shows the recorded incident, reflected and transmitted stress waves when the input stress wave has an amplitude of 406 MPa. From Table 2, we can observe that the density and the longitude wave velocity of the bar in the BPM agree well with those of the steel material. Therefore, we can use micro parameters in Table 2 to simulate the steel bars.

From Figure 4a, it can be seen that the transmitted waveform through the sample is an approximately half-sinusoidal wave. However, the reflected wave presents a more complex waveform. A platform appears on the reflected waveform. When the time is over around 540 μs, the platform gradually fades away and the waveform drops down to a valley. After that, the waveform suddenly increases instead, which was induced by the failure of the sample. Hence, the turning point on the reflected waveform resulted from the macro failure of the sample. These phenomena have a good consistency with laboratory observation by [8], which indicates that our model is feasible to simulate SHPB tests on the brittle rock. Figure 4b shows that the summed waveform between the incident and reflected waveforms basically overlaps with the transmitted waveform, which verifies that stresses at the front and rear interfaces of the sample keep balance. According to [8], the platform presents an approximate constant strain rate.

**Figure 5:**
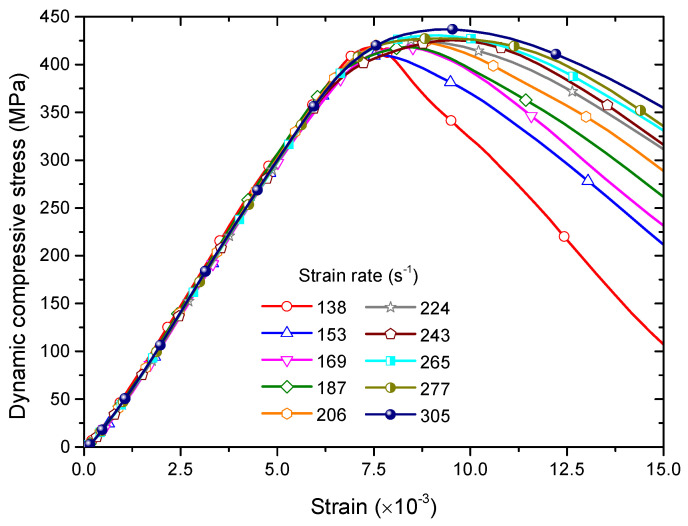
Dynamic stress–strain curves of the intact sample under different strain rates. For interpretation of the references to color in this figure, the reader is referred to the electronic version of this paper.

**Figure 6:**
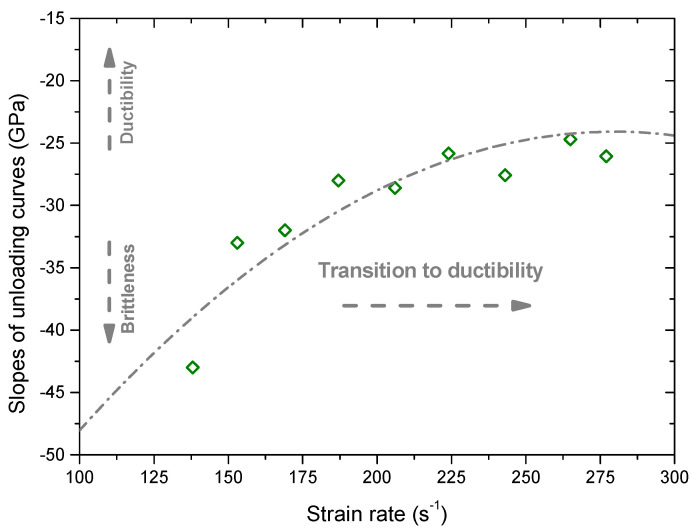
Slope of the post-peak stress–strain curve versus the strain rate.

Figure 5 shows dynamic stress–strain curves of the intact sample under different strain rates. It can be seen that the stress basically increases with the increment of the strain in a linear manner before the peak stress. After the peak stress, the stress decreases with the increment of the strain. When the strain rate is equal to 138 s^−1^, the post-peak stress–strain curve decreases in a relatively abrupt manner. With the increase of the strain rate, it begins to decrease gently with the strain. When the strain rate is equal to 305 s^−1^, the post-peak stress–strain curve decreases very gently with the strain. It also can be seen that the peak stress (dynamic compression strength) increases with the increase of the strain rate. To quantitatively characterize this unloading behavior, we calculated the slope of the post-peak curve which can be used as the brittleness index. Figure 6 shows the slope of the post-peak curve versus the strain rate. It first abruptly increases with the increase of the strain rate, then its variation become gentle. We can infer that there exists a transition from the brittle to ductile failure with increasing the strain rate.

**Figure 7:**
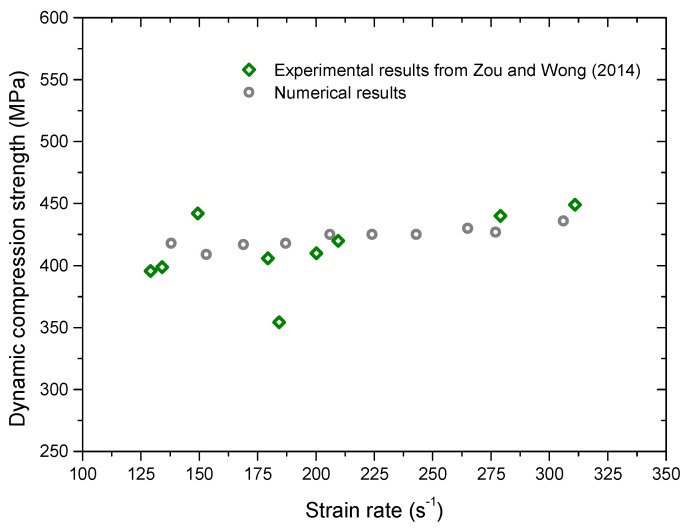
Comparison between the dynamic compression strength by numerical simulation and the experimental result by [20]. For interpretation of the references to color in this figure, the reader is referred to the electronic version of this paper.

Figure 7 shows the comparison between the dynamic compression strength and the experimental results obtained by [20]. We can observe that the numerical results have an increasing trend with the increment of the strain rate. Values of numerical results match very well with those of experimental results. It indicates that micro parameters in Table 3 can be used to simulate the dynamic compression behavior of the Carrara marble.

**Figure 8:**
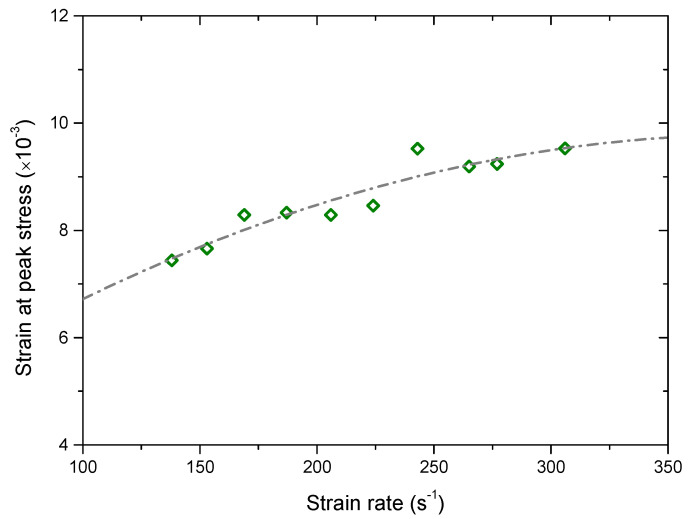
Variation of the strain at the peak stress versus the strain rate.

Figure 8 shows the variation of the strain at the peak stress versus the strain rate. It can be seen that the strain at the peak stress has an increasing trend as the strain rate increases. We can infer that the capability of the sample to tolerate the maximum strain increases with the strain rate increasing. To some extent, this also reflects a transition from the brittle to ductile failure of the sample.

**Figure 9:**
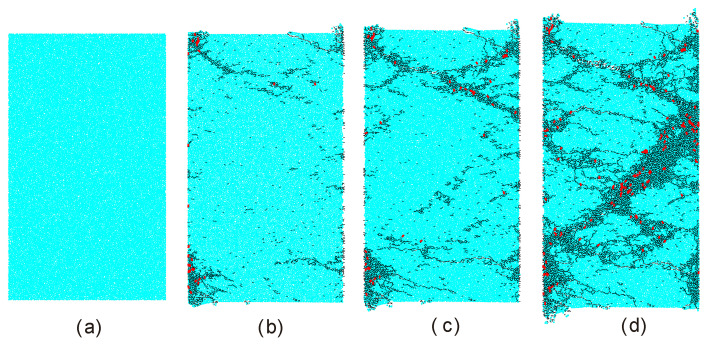
Progressive failure process of the intact sample under a dynamic compressive strain of 169 s^−1^. (**a**) the first stage; (**b**) the second stage; (**c**) the third stage; (**d**) the fourth stage. The fourth stage shows the state in which the macro failure formed. With the compression strain increasing, the sample could gradually become thinner. The black and red denote the tensile and shear micro cracks respectively. For better presentation, red shear cracks were thickened. For interpretation of the references to color in this figure, the reader is referred to the electronic version of this paper.

Figure 9 shows the progressive failure process of the intact sample under a compressive strain rate of 169 s^−1^. The progressive failure process was divided into four stages. In the first stage, the compressive stress keeps increasing but no micro cracks were produced. In the second stage, the local stress exceeds the bond strength of the contact. There are many micro cracks near the lower and upper boundaries while only a few micro cracks appear near the center of the sample. In the third stage, micro cracks near the lower and upper boundaries coalesce into several spalling macro cracks. Also, there are a considerable number of micro cracks produced near the center of the sample. The sample begins to be split into fragments by spalling macro cracks near the lower and upper boundaries. In the fourth stage, macro spalling cracks are obviously formed as well as an oblique fracture zone. It can be also observed that the number of tensile micro cracks (black) is always far larger than that of shear micro cracks (red). However, the proportion of the shear micro cracks seems to increase in the fourth stages.

**Figure 10:**
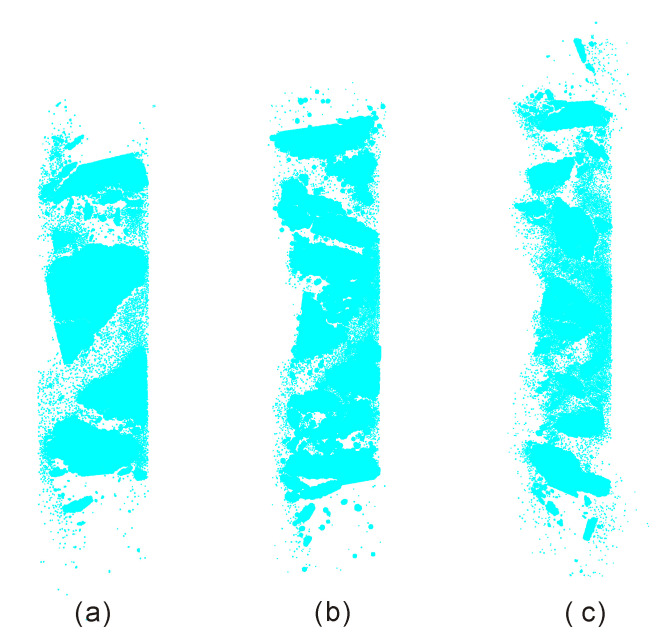
Final failure configurations of the sample after loaded by compressive strain rates of (**a**) 138 s^−1^; (**b**) 169 s^−1^; (**c**) 206 s^−1^. All failed samples were separated to the incident and transmitted bars and cannot be further loaded.

Figure 10 shows final failure configurations of the intact sample after loaded by different compressive stain rates. It can be seen that all samples were split into fragments by spalling macro cracks. With the increase of the compressive strain rate, the number of fragments increases while its size decreases. We can infer that the total number of micro cracks should increases with the compressive strain rate.

**Figure 11:**
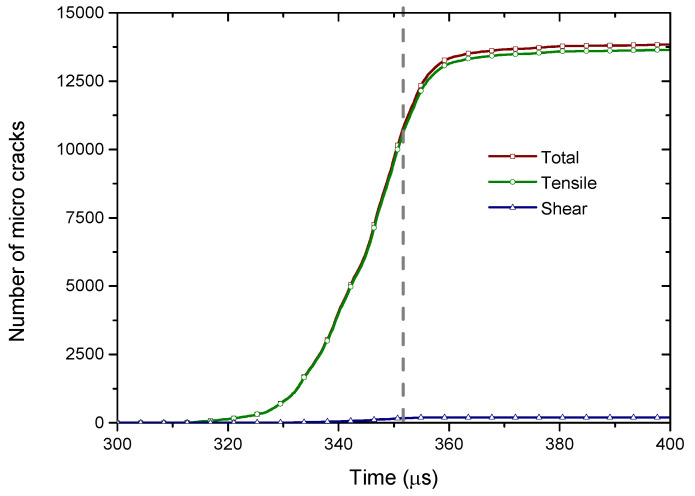
Variation of the micro crack number in the sample with time when the sample was compressed under a strain rate of 206 s^−1^. For interpretation of the references to color in this figure, the reader is referred to the electronic version of this paper.

Figure 11 shows the variation of the micro crack number in the sample versus time when the compressive strain is 206 s^−1^. It can be seen that numbers of the total and tensile micro cracks have a similar variation trend with time. That is, each micro crack number first increases gently with time, then it abruptly increases and at last it basically keeps a constant. Generally, the number of shear micro cracks is far smaller than that of the tensile micro cracks. When the time is over around 352 μs (the gray vertical dash line), the number of shear micro cracks basically keeps invariant, while the number of tensile micro cracks still increases to around 13,750 and then it changes gently. It indicates that the formation of the shear micro cracks was restricted when the number of the tensile micro cracks reaches a critical threshold.

**Figure 12:**
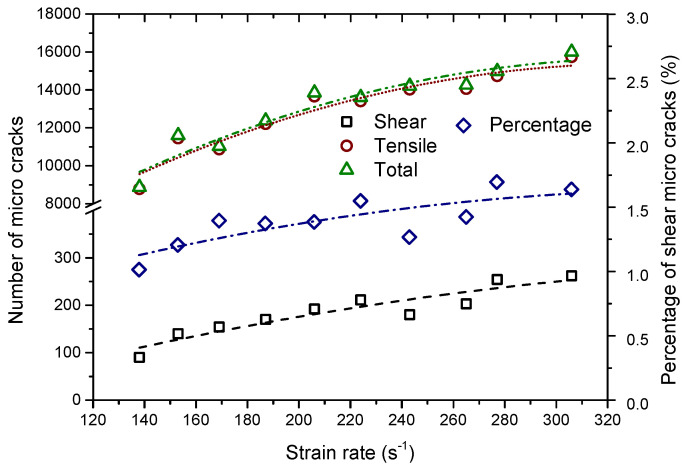
Variation of micro crack numbers for the completely failed sample versus the strain rate, i.e., the total, tensile, and shear micro cracks, respectively. The percentage variation of shear micro cracks versus the strain rate was also plotted together.

Figure 12 displays the variation of micro crack numbers for the completely failed sample versus the compressive strain rate—i.e., the total, tensile, and shear micro cracks, respectively. Meanwhile, the percentage variation of the shear micro crack versus the compressive strain rate was plotted together. It can be observed that numbers of the total, tensile, and shear micro cracks nonlinearly increase with increasing the compressive strain rate. When the strain rate is less than around 220 s^−1^, both the number of the total and tensile micro cracks abruptly increases with the strain rate increasing. After that, these two numbers increase relatively gently with the increment of the strain rate. However, shear micro cracks always increase in number more gently with the strain rate compared with tensile micro cracks. The percentage of the shear micro cracks increases as the strain rate increases. Namely, there exists a transition trend from tensile to shear failure in the microscopic scale as the strain rate increases.

### 3.2. Case of the Sample Containing An Open Flaw

**Figure 13:**
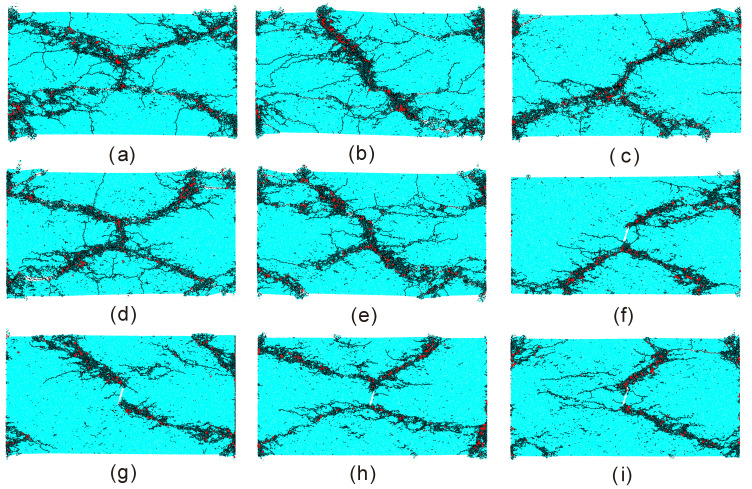
Final failure configurations of nine different samples with an open flaw with the incline angle of 15° when the random number seed is (**a**) 106; (**b**) 136; (**c**) 156; (**d**) 216; (**e**) 266; (**f**) 386; (**g**) 396; (**h**) 406; (**i**) 436, respectively. The average of the compressive strain rates is around 120 s^−1^. The black and red denote the tensile and shear micro cracks, respectively. For better presentation, red shear cracks were thickened. For interpretation of the references to color in this figure, the reader is referred to the electronic version of this paper.

In this section, we mainly investigate the progressive failure process of the sample containing an open flaw under different compressive strain rates. As mentioned above, the sample is an assembly made up of particles of which radius and position were randomly distributed. In PFC-based BPM, a given random number seed can generate a specific model. Results considering different random number seeds often have some differences with each other. However, the general or overall characteristics of these results should be similar. Figure 13 shows the final failure configurations of nine samples containing an open flaw under an average compressive strain rate of 120 s^−1^ when the incline angle is 15°. It can be seen that there are at least two macro cracks produced in each sample. These macro cracks mainly consist of tensile micro cracks while the shear micro crack is relatively invisible. Basically, all the macro tensile cracks strike from the flaw tips to the corners of the sample as a diagonal mode although their morphologies show some differences. Not all the diagonal macro cracks can be mobilized. When four diagonal macro cracks were produced simultaneously, an ‘X’-shape failure configuration can be ultimately captured.

**Figure 14:**
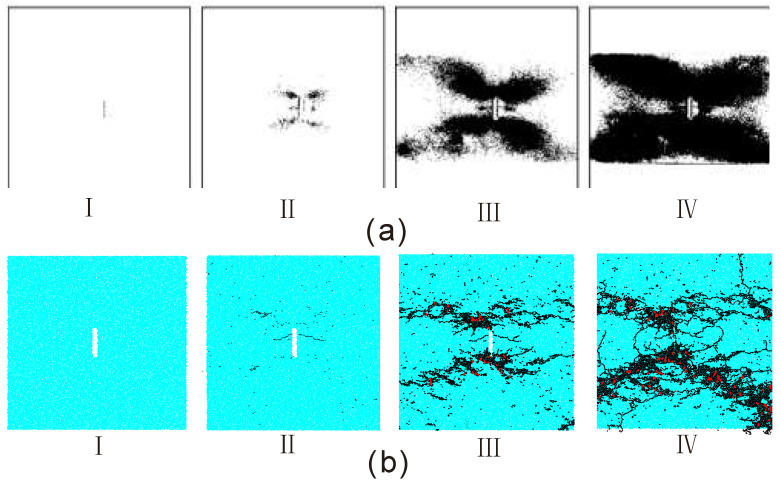
Comparison of the progressive failure process of the sample containing an open flaw with the incline angle of 0° under the compressive strain rate of 121 s^−1^. (**a**) the experimental results by [20]; (**b**) the numerical results by BPM. The black and red denote the tensile and shear micro cracks respectively. For better presentation, red shear cracks were thickened. For interpretation of the references to color in this figure, the reader is referred to the electronic version of this paper.

We compared the ‘X’-shape failure process with that of the experiment result by [20]. From Figure 14a, the experimental progressive failure process of the sample containing an open flaw can be divided into four stages. In the first stage, the stress inside the sample kept increasing while there were no micro cracks produced. In the second stage, four clustering zones of the micro cracks symmetrically appear at two flaw tips and the middle part of the flaw. Four zones corresponding to two flaw tips initially form an ‘X’-shape failure configuration. In the third stage, the propagation of the early-formed macro crack near the midpoint of the flaw was prohibited while four zones of the micro cracks near the two flaw tips become longer and wider which basically developed along the diagonal line. In the fourth stage, four zones of the micro cracks are very prominent which take up most of the sample area. From Figure 14b, the numerical simulation results by BPM for each stage agree well with the experiment results. Additionally, we can readily clarify that the tensile micro cracks play the domain role in the progressive failure process of the sample containing an open flaw. Whereas this is often difficult to realize in the experiments.

**Figure 15:**
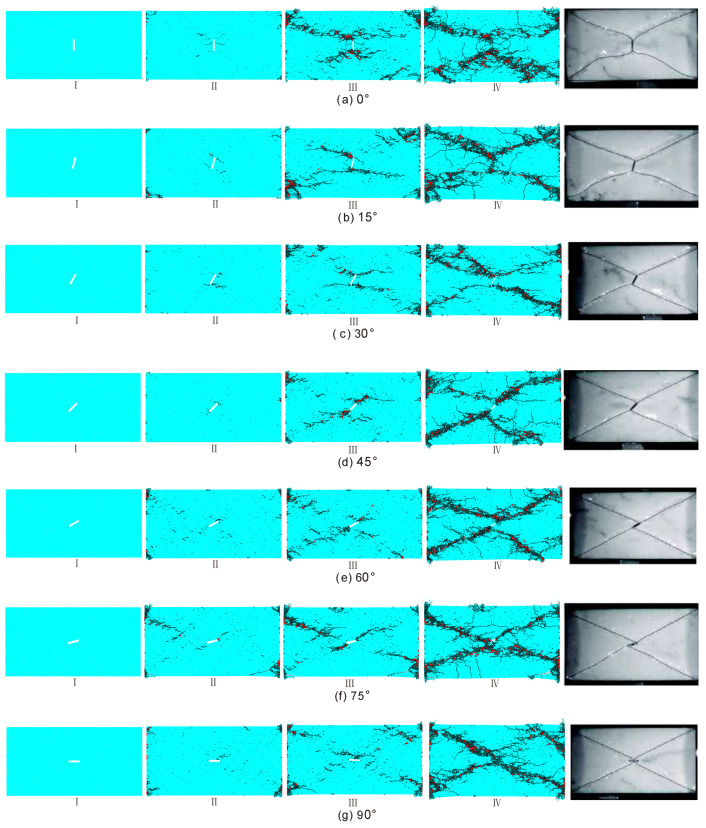
Progressive failure processes of samples containing an open flaw under dynamic compression when (**a**) α = 0°, $\dot{\varepsilon }$ = 121 s^−1^; (**b**) α = 15°, $\dot{\varepsilon }$ = 123 s^−1^; (**c**) α = 30°, $\dot{\varepsilon }$ = 119 s^−1^; (**d**) α = 45°, $\dot{\varepsilon }$ = 118 s^−1^; (**e**) α = 60°, $\dot{\varepsilon }$ = 118 s^−1^; (**f**) α = 75°, $\dot{\varepsilon }$ = 122 s^−1^; (**g**) α = 90°, $\dot{\varepsilon }$ = 119 s^−1^. The black and red denote the tensile and shear micro cracks respectively. Rightmost pictures are experimental results obtained by [20]. For better presentation, red shear cracks were thickened. For interpretation of the references to color in this figure, the reader is referred to the electronic version of this paper.

Figure 15 shows the progressive failure processes of samples containing an open flaw with varied incline angle under dynamic compression. For each case of the incline angle, four stages were considered. Also, the final failure configurations were compared with the experimental results by [20]. For the first stage, it mainly involves in the stress increase inside the sample and occurrence of a few micro cracks.

For the second stage, micro cracks begin to appear for all cases of the incline angle. These micro cracks are mainly tensile cracks and lack of shear ones. The crack initiation position and formed macro cracks depend on the incline angle. When the incline angle is 0°, the micro cracks mainly appear at two flaw tips and near the midpoint of the flaw. Basically, these micro cracks symmetrically develop and gradually form macro cracks, e.g., the two in the middle of the flaw are typical macro tensile macro cracks. The four macro cracks tend to form a larger ‘X’-shape failure zone. When the incline angle is 15°, there are a pair of anti-wing cracks initiating from the two flaw tips. Different from the case of 0°, very few micro cracks appear near the midpoint of the flaw. As the incline angle ranges from 30° to 75°, the micro cracks mainly initiate near the flaw tips. When the incline angle is 90°, the micro cracks initiate near the midpoint of the flaw.

For the third stage, shear micro cracks (red) commonly appear mixed with the tensile micro cracks while the number is still much smaller than that of the tensile crack. The propagation of macro cracks formed in the second stage are prohibited, i.e., the tensile macro cracks near the midpoint of the flaw (0°), the anti-wing macro cracks at two flaw tips (15°). As the incline angle ranges from 0° to 45°, the cracks mainly develop from the flaw to four corners of the samples. When the incline angle is larger than 60°, the cracks could develop from four corners to the flaw. In this stage, the diagonal trajectories of the macro cracks begin to emerge on the whole.

For the fourth stage, the micro cracks inside all samples were coalesced into persistent diagonal macro cracks. The proportion of the shear micro cracks increases. Although the macro cracks present a similar ‘X’ shape, there exist some differences for the cracking mode. When the incline angle is smaller than 45°, four diagonal macro cracks have flexural trajectory which are basically symmetrical to the vertical line. With the incline angle increasing, the flexure gradually fades away. When the incline angle is larger than 45°, the NE-striking macro cracks are basically along the diagonal lines of the rectangle while two other macro cracks are very typical anti-wing macro cracks. The straightness of the macro cracks increases with increasing the incline angle. When the incline angle is 90°, anti-wing macro cracks disappear, and the ‘X’-shape failure zones consist of four macro cracks of which each one strikes from the position near the midpoint to the corner of the sample. From Figure 15, we can observe that our results of the final failure configurations for different incline angles are very consistent with the experimental results obtained by [20] including the cracking mode and the trajectory development of the macro cracks.

**Figure 16:**
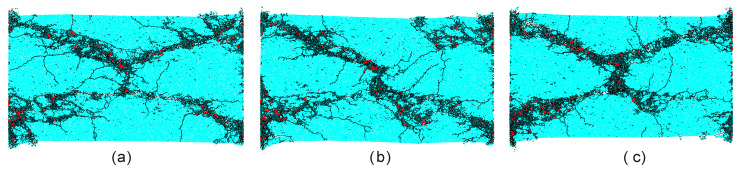
Failure configurations of the sample containing an open flaw with the incline angle of 15° under compressive strain rates of (**a**) 123 s^−1^; (**b**) 152 s^−1^; (**c**) 175 s^−1^. The black and red denote the tensile and shear micro cracks respectively. For better presentation, red shear cracks were thickened. For interpretation of the references to color in this figure, the reader is referred to the electronic version of this paper.

Figure 16 displays final failure configurations of samples containing an open flaw with the incline angle of 15° when the compressive strain rate varies. It can be observed that failure configurations for different strain rates are very similar and all behave as an ‘X’ shape. It indicates that the macro failure configuration of the flaw sample is rate-independent within this scope of the strain rate.

## 4. Discussion

**Figure 17:**
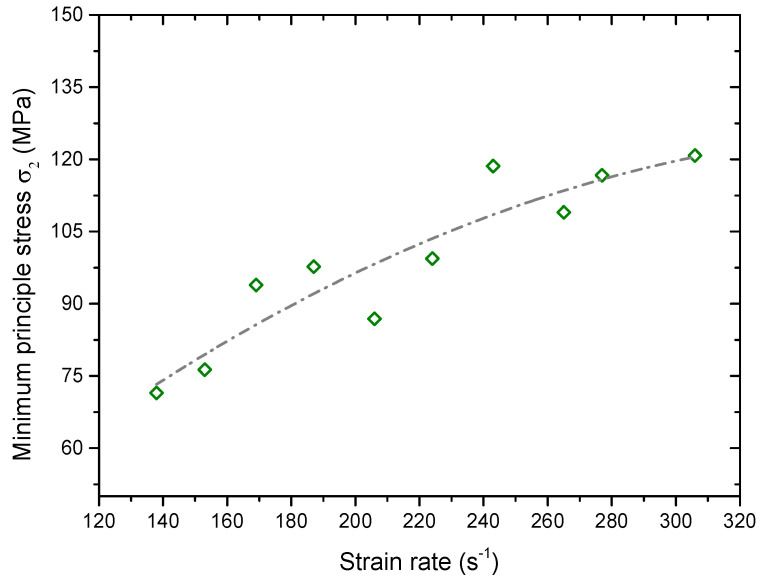
Variation of the peak of the *σ_2_(t)* versus the strain rate.

During the simulation, we set a measured circle with a radius of 7.5 mm in the intact sample. The center of the circle overlaps with that of the sample. Via this measure circle, we can monitor the maximum principle stress *σ*_1_(*t*) (along the loading direction) and the minimum principle stress *σ*_2_(*t*) (normal to the loading direction) in the sample. According to the recorded signal, we found that *σ*_2_(*t*) was a compressive stress pulse which was induced by the stress wave passing through the sample. Thus, *σ*_2_(*t*) has an effect of confinement. Figure 17 shows the peak of the *σ*_2_(*t*) versus the strain rate, which increases nonlinearly as the strain rate increases. It indicates that the effective confined pressure increases. We can now understand that there exists a transition of the failure for the sample from the brittleness to the ductility with increasing the strain rate.

It was found that the deviatoric stress was related to the shear failure of the material. The deviatoric stress *σ_d_*(*t*) in the measure circle can be calculated as

(5) $$\sigma_{d}(t)=\sigma_{1}(t)-\sigma_{2}(t),$$

**Figure 18:**
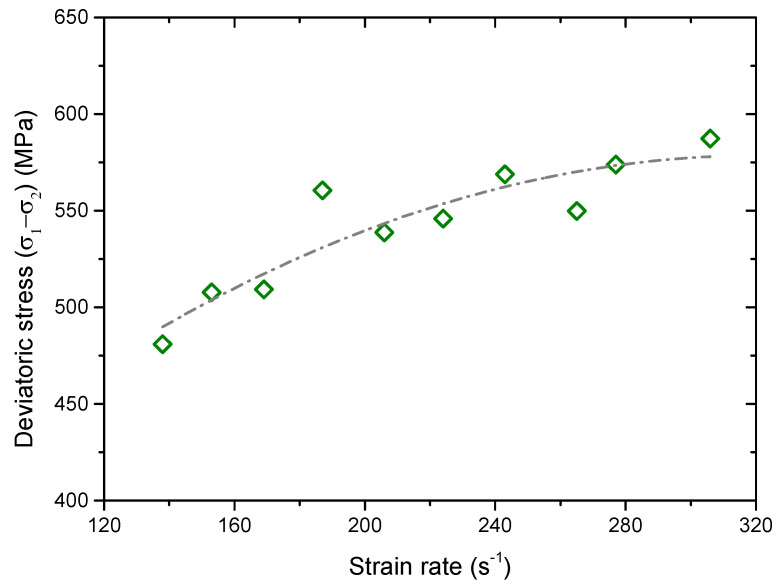
Maximum deviatoric stress versus the strain rate.

From the recorded signal, *σ_d_*(*t*) was also a pulse which was induced by the stress wave passing through the sample. Figure 18 shows the peak of *σ_d_*(*t*) versus the strain rate. It can be seen that the value increases as the strain rate increases. Thus, we can now understand that the number of shear micro cracks and its percentage in total micro cracks increases with the strain rate increasing (see Figure 12). The reason for this phenomenon may result from the uncoordinated deformation in the sample. The dynamic loading with a strain rate ~10^2^ s^−1^ is an extremely rapid process. Different from the static loading, particles in the sample under this strain rate do not have enough time to finish deformation adjustment to achieve a balance state. This unbalance state may induce uncoordinated deformation and stress concentration in the sample. With the increase of the strain rate, the time in which particles adjusted deformation was further shortened. The deviatoric stress related to the uncoordinated deformation thus increases with the strain rate. Meanwhile, the increase of the uncoordinated deformation may produce more local stress concentration areas, which makes more cracking areas form in the sample. Hence, the number of fragments increases as the strain rate increases (see Figure 10).

**Figure 19:**
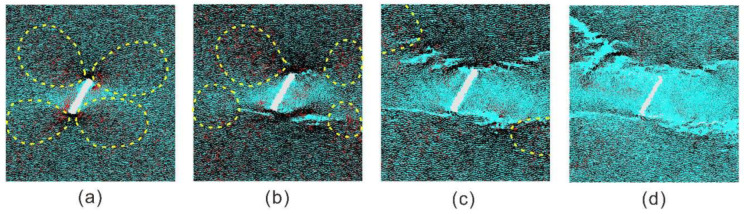
Contact force chains inside the flawed sample during the progressive failure process. (**a**) the first stage; (**b**) the second stage; (**c**) the third stage; (**d**) the fourth stage. The black and red denote the compressive and tensile force, respectively. The magnitude of the force is proportional to the thickness. For interpretation of the references to color in this figure, the reader is referred to the electronic version of this paper.

In the BPM, the bond will be broken when the contact tensile stress exceeds the tensile bonded strength. Hence, the contact force in the sample was analyzed to reveal the mechanism of the final ‘X’-shape failure configuration. Figure 19 shows distributions of compressive and tensile force chains corresponding to four stages of progressive failure. In the first stage, there are four concentration areas of the contact force near two flaw tips, which were enclosed by yellow dashed curves. It can be seen that both the compressive force chain and the tensile force chain in each enclosed area is much denser and has wider thickness compared with other areas away from the flaw tip. The red force chains in four enclosed areas basically have similar thickness. This indicates that tensile stresses in four enclosed areas basically have the similar magnitudes and the contact bond in four enclosed areas can be broken simultaneously. Therefore, tensile cracks can be observed near four corners of two flaw tips in Figure 15. As the dynamic compressive stress increased, the concentration of the contact force in four enclosed areas strengthened in the second stage. When the tensile or shear loads exceeded the bonded strength of the contact, considerable tensile micro cracks were produced. The cracking of the contact bond released the concentration of the contact force locally and also moved the concentration area. In the third stage, the tensile force in four concentration areas all induced tensile micro cracks, which formed ‘X’-shape macro cracks. We can see that four stress concentration areas were obviously released, especially for the lower left and the upper right areas due to more micro cracks. In the fourth stage, micro cracks accumulated to form an ‘X’-shape final failure configuration. Four stress concentration areas were completely released. In the ‘X’-shape band, both the tensile and compressive contact force chain are relatively less. Meanwhile, these two types of contact force chain basically distribute in a uniform manner. We also investigated other cases considering varied incline angles of the flaw and varied strain rates and found similar four concentration areas and evolution modes of the contact force. On this basis, we can now understand that samples containing an open flaw with varied incline angle all could produce a similar ‘X’-shape final failure configuration under dynamic compression.

## 5. Conclusions

Understanding failure characteristics of the brittle rock under high-strain-rate loads such as the strong earthquake and engineering blasting is of great importance for the practical rock engineering. The dynamic-induced failure of the brittle rock is a progressive process while its mechanism still needs to be clarified. This paper investigated the progressive failure characteristics of the brittle rock under SHPB high-strain-rate compression using the BPM from the micromechanical view of the point. The intact sample and the sample containing an open flaw with varied incline angles were considered respectively. Some important conclusions were reached.

The intact sample first experienced in microcracking, then crack coalescing, and finally splitting into fragments. The total number of micro cracks, the percentage of shear micro cracks, the number of cracked fragments, the dynamic compressive strength, and the strain at peak stress all increased with the strain rate increasing. There existed a transition trend from the tensile to shear microscopic failure in the sample and a transition for the failure from brittleness to ductility with increasing the strain rate.

For the flawed sample, the microcracking position and the types of the formed macro cracks depended on the incline angle of the flaw in the initial stage. However, propagation of these early-formed macro cracks were prohibited in the later stages. New micro cracks were produced and then coalesced into diagonal macro cracks which could all form ‘X’-shape failure configuration regardless of the incline angle of the flaw. We explored micromechanics on progressive failure characteristics of the brittle rock under dynamic loads.

Our study results can provide some scientific basis for practical engineering, such as the shale gas development and the directional blasting engineering. For the gas shale engineering, it is crucial to induce tensile fractures in the reservoir as many as possible by hydraulic fracturing. The progressive failure process of the shale under dynamic loads could produce considerable micro cracks compared with the static case, that is, the dynamic hydraulic fracturing. On the other hand, the dynamic ‘X’-shape failure independent on the flaw angle has an important guiding significance on the directional blasting engineering. Our current study could potentially give some technical and scientific support in practical engineering.

## Figures and Tables

**Table 1:** Calibrated micro parameters of steel bars.

Particle Parameters		Parallel Bond Parameters	
Contact Young’s modulus ($E_{c}$) (GPa)	206	Young’s modulus of the parallel bond (${\bar{E}}_{c}$) (GPa)	206
Ratio of normal to shear contact stiffness ($k_{n}/k_{s}$)	2	Ratio of normal to shear stiffness of the parallel bond (${\bar{k}}_{n}/{\bar{k}}_{s}$)	2
Contact friction coefficient (μ)	0.8	Tensile strength of the parallel bond (${\bar{\sigma }}_{c}$) (MPa)	10^8^
Ratio of maximum to minimum particle radius (*R*_max_/*R*_min_)	1	Shear strength of the parallel bond (${\bar{\tau }}_{c}$) (MPa)	10^8^
Minimum particle radius (*R*_min_) (mm)	2	Radius multiplier ($\bar{\lambda }$)	1
Particle density (*ρ*) (kg/m^3^)	8804		

**Table 2:** Comparisons on macro properties between the BPM and the steel bar.

Physical Properties	Steel	BPM
Density *ρ* (kg/m^3^)	7894	7894 (continuum-equivalent)
Longitude wave velocity *C* (m/s)	5048	5053

**Table 3:** Calibrated micro parameters of the Carrara marble.

Particle Parameters		Parallel Bond Parameters	
Contact Young’s modulus ($E_{c}$) (GPa)	60	Young’s modulus of the parallel bond (${\bar{E}}_{c}$) (GPa)	60
Ratio of normal to shear contact stiffness ($k_{n}/k_{s}$)	1.8	Ratio of normal to shear stiffness of the parallel bond (${\bar{k}}_{n}/{\bar{k}}_{s}$)	1.8
Contact friction coefficient (μ)	0.8	Tensile strength of the parallel bond (${\bar{\sigma }}_{c}$) (MPa)	350 ± 80
Ratio of maximum to minimum particle radius (*R*_max_/*R*_min_)	1.6	Shear strength of the parallel bond (${\bar{\tau }}_{c}$) (MPa)	550 ± 80
Minimum particle radius (*R*_min_) (mm)	0.08	Radius multiplier ($\bar{\lambda }$)	1.0
Particle density (*ρ*) (kg/m^3^)	3228		
